# Post-Myocardial Infarction Ventricular Septal Defect Successfully Treated with Impella as Bridge to Cardiac Transplantation

**DOI:** 10.1155/2022/5690844

**Published:** 2022-07-31

**Authors:** Lauren Giudicatti, Benjamin Silbert, Xiao-Fang Xu, Anthony Putrino, Felicity Lee, Amit Shah, Robert Larbalestier, Karim Slimani, Andrew Laycock, Kaitlyn Lam

**Affiliations:** ^1^Department of Cardiology, Fiona Stanley Hospital, Perth, Australia; ^2^Advanced Heart Failure and Cardiac Transplantation Unit, Fiona Stanley Hospital, Perth, Australia; ^3^Department of Cardiothoracic Surgery, Fiona Stanley Hospital, Perth, Australia; ^4^PathWest, Fiona Stanley Hospital, Perth, Australia

## Abstract

A 63-year-old female presented late with anterior ST-elevation myocardial infarction and cardiogenic shock. This was complicated by acute ventricular septal defect with large left-to-right shunt. An Impella CP was inserted on day seven with rapid haemodynamic improvement. This facilitated bridge to cardiac transplant on day twelve post-MI.

## 1. History of Presentation

A 63-year-old female presented following several days of central chest pain and dyspnoea. Past medical history was remarkable for mild intermittent asthma, ex-smoking status, and overweight body habitus (82 kg, body mass index 27). 12-lead electrocardiogram showed anterior ST-segment elevation myocardial infarction (MI). The patient underwent urgent coronary angiography. The left anterior descending artery (LAD) was occluded with ostial thrombus. Revascularisation of the LAD was performed with Xience Sierra 3.5 × 33 mm drug-eluting stent. Slow flow improved following intracoronary eptifibatide. A 50 cc intra-aortic balloon pump (IABP) was inserted for persistent hypotension at full augmentation. Intravenous heparin and eptifibatide infusions were continued. The patient returned to the coronary care unit for routine post-MI management.

## 2. Differential Diagnosis and Investigations

**Figure 1:**
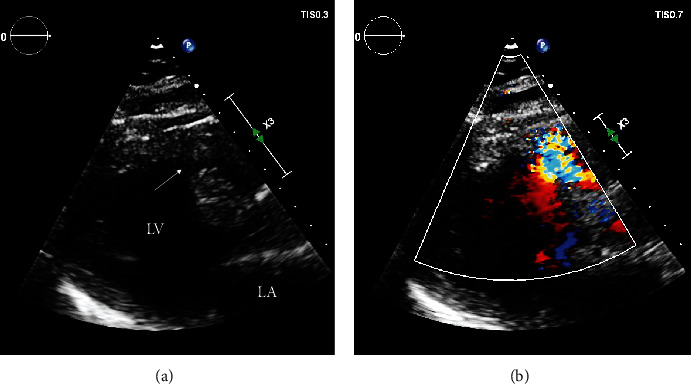
Transthoracic echocardiogram, parasternal long axis view, demonstrating a 1.3 cm muscular VSD in the basal anteroseptum ((a), arrow) with left-to-right shunting on colour Doppler (b).

Transthoracic echocardiogram (TTE) on day two demonstrated 1.3 cm ventricular septal defect (VSD) at the basal anteroseptum with a large eccentric shunt from left ventricle (LV) to right ventricular (RV) outflow tract (Figure 1). The LV ejection fraction was 40% with akinesis of the interventricular septum (IVS) and LV apex on mechanical and inotropic support. On day five, trial of IABP removal failed due to rapid deterioration into cardiogenic shock and multiorgan failure, requiring prompt reinsertion. Right heart catheterisation was performed on day six. A significant step-up in oxygen saturation was demonstrated between RV (74%) and pulmonary artery (88%), and calculated shunt fraction (Qp : Qs) was 3.4, consistent with a haemodynamically significant shunt. Pulmonary capillary wedge pressure and transpulmonary gradient were elevated at 21 mmHg and 15 mmHg, respectively.

## 3. Management

**Figure 2:**
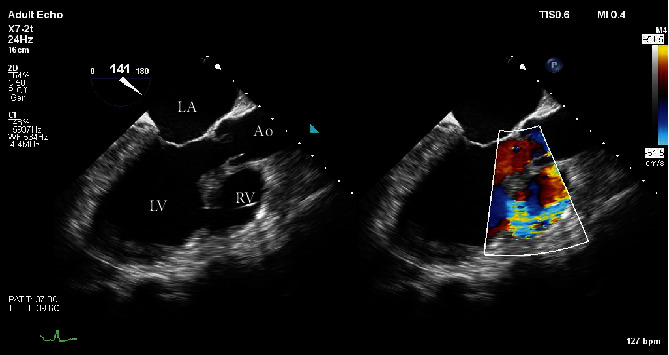
Transesophageal echocardiogram, midesophageal 3-chamber view taken whilst on Impella support, showing large VSD with left-to-right shunting on colour Doppler.

The patient developed refractory cardiogenic shock despite IABP support and escalating inotrope and vasodilator therapy. Following heart team meeting, a decision was made to insert a percutaneous microaxial flow pump (Impella) as a bridge to either VSD repair at approximately six weeks post-MI, with or without left ventricular assist device (LVAD), or urgent cardiac transplantation. Urgent insertion of an Impella CP was performed on day seven post-MI via surgical cut down to the left axillary artery. The higher power Impella 5 or Impella 5.5 was not available at the time of insertion. The patient experienced rapid haemodynamic improvement within minutes and complete wean of inotropic/IABP support within hours which was sustained over the following days. Transesophageal echocardiogram (TEE) and continuous SvO2 monitoring were used to guide manipulation of Impella flow and shunt fraction, to good effect (Table 1).  Figure 2 demonstrates persistent left-to-right shunt on TEE following Impella insertion.

**Figure 3:**
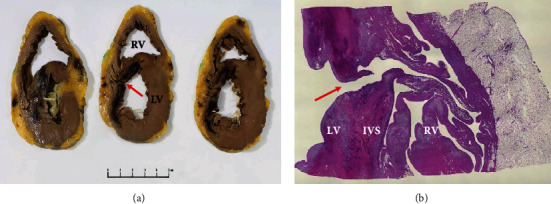
Explanted heart showing. (a) Cross-section through the basal (left) to apical (right) left ventricle (LV) demonstrating haemorrhagic infarct involving the interventricular septum (IVS) and anterior LV (asterisk). A defect is evident through the infarcted region of the IVS (arrow). (b) At low power, a full thickness defect is evident through the region of infarction (arrow), allowing communication from LV to RV, via a tortuous path.

The patient had no contraindications to cardiac transplant and was urgently listed. A suitable donor became available on day five post-Impella insertion (12 days post-MI). Total donor ischemia time was 100 minutes. Immunologic matching profile was favourable. Macroscopic and microscopic examinations of the explanted heart are shown in Figure 3. The patient was extubated on day five.

## 4. Follow-Up

The patient experienced a postoperative stroke requiring inpatient rehabilitation and was discharged home on day 47 with residual right foot drop.

## 5. Discussion

Acute ischaemic VSD is a rare but catastrophic mechanical complication of acute MI [1, 2]. Post-MI VSDs are typically diagnosed between 3 and 7 days postinfarct, although it can occur as early as 24 hours [3]. Contemporary management of MI with early revascularisation has led to a reduction in prevalence to less than 1% [2, 3]. Despite this, untreated mortality remains over 90% at 30 days [2, 3]. With intervention, mortality is reduced to approximately 65% at 30 days [4].

Timing of repair for post-MI VSD remains a major challenge. Data from the Society of Thoracic Surgeons National Database (*n* = 2876) demonstrated a reduction in mortality from 54% to 18% with early (<7 days) versus delayed (7 days or later) surgical repair, respectively [5]. The durability of early surgical is largely hindered by friable peri-infarct tissue which may cause incomplete closure and residual shunting in up to 20% of patients [3]. Transcatheter repair provides a less invasive alternative where surgical repair is contraindicated, although it is also limited by defect location and peri-infarct tissue quality [3]. Where suitable, it may be used either as a bridge to haemodynamic stability and definitive surgical repair, or as a definitive therapy in itself [4].

Temporary mechanical circulatory support (MCS) is increasingly utilised in patients with cardiogenic shock post-MI VSD to reduce left-to-right shunting and augment cardiac output [6]. This allows time for the scar to consolidate and improves likelihood of durable repair. The optimal timing and method of MCS remain contentious [6]. Thiele and colleagues describe the use of IABP in 23 patients as a bridge to early or delayed surgical repair [4, 7]. Peri-operative mortality remained high at 83% and 57%, respectively, and later studies have failed to show any significant mortality benefit [4, 7]. Peripheral veno-arterial extracorporeal membrane oxygenation (VA-ECMO) provides more complete circulatory support compared to IABP, with more favourable outcomes when used as a bridge to reparative surgery [8]. Limitations include a high risk of complications, insufficient LV unloading, and worsening of left-to-right shunting [6, 8]. Finally, LVAD has been used with success in isolated VSD cases as a longer term MCS option, both at the time of surgical repair and following absence of postoperative myocardial recovery [6].

In recent years, the Impella intracardiac microaxial pump (Abiomed Danvers, Massachusetts) has gained popularity as a less invasive and more durable alternative to traditional MCS strategies following acute MI [9]. The Impella offers superior reduction in wedge pressure and minimisation of left-to-right shunting compared to IABP, with conceivable benefit for patients with VSD, although it remains off-label for this indication [4, 6, 9]. The Impella CP provides a maximum output of approximately 3.7 L/minute [9]. In patients with VSD, excessive pump power and flow may result in right to left shunting and subsequent hypoxia, and careful manipulation with a goal of maintaining saturations above 92% is therefore required. In our case, after several days of minimal improvement on IABP, we observed a dramatic haemodynamic response and rapid reversal of end organ damage following Impella CP insertion. This reflects the superior augmentation of cardiac output provided by the Impella. Isolated case reports have previously shown similar success with Impella in post-MI VSD [10]. Patanè and colleagues have described the use of the Impella Recovery as a bridge to transplantation in a post-MI VSD unsuitable for surgical repair [10]. The importance of timely implantation of Impella before irreversible haemodynamic deterioration is again emphasised in our case.

## 6. Conclusion

Post-MI VSD is an important and life-threatening complication of acute MI. Conventional MCS options including IABP and VA-ECMO can help achieve temporary haemodynamic stability where delayed surgical intervention is preferable, but may be limited by incomplete LV unloading and high risk of complications. We have demonstrated that the Impella is an effective alternative bridge to transplantation in patients with VSD and refractory cardiogenic shock. Future experience with this device in acute post-MI VSD will help form treatment pathways.

## 7. Learning Objectives

1. To use a multidisciplinary and haemodynamic-based approach to determine optimal timing of intervention in patients with acute post-MI VSD and refractory cardiogenic shock.
2. To understand the role of mechanical circulatory support in providing a bridge to definitive therapy in the setting of acute post-MI VSD.

## Figures and Tables

**Table 1:** Haemodynamics and biochemistry pre versus post-Impella insertion demonstrating rapid improvement without increase in right-to-left shunting or right heart pressures.

**Variable**	**Baseline (on IABP)**	**<6-hours post-Impella**	**24-hours post-Impella**
CVP	20	10	12
PCWP	21	23	23
PASP	45	49	42
CI	3.3	2.8	3.5
SvO2	43	84	76
Lactate	1.1	1.1	0.8
Creatinine	172	200	99

IABP: intra-aortic balloon pump; CVP: central venous pressure; PCWP: pulmonary capillary wedge pressure; PASP: pulmonary artery systolic pressure: CI: cardiac index; SvO2: oxygen saturation in venous blood.

## Abbreviations

MI: Myocardial infarction
LAD: Left anterior descending artery
IABP: Intra-aortic balloon pump
TTE: Transthoracic echocardiogram
VSD: Ventricular septal defect
LV: Left ventricle
RV: Right ventricle
IVS: Interventricular septum
LVAD: Left ventricular assist device
TEE: Transesophageal echocardiogram
MCS: Mechanical circulatory support
VA-ECMO: Veno-arterial extracorporeal membrane oxygenation.
